# ULMR: An Unsupervised Learning Framework for Mismatch Removal

**DOI:** 10.3390/s22166110

**Published:** 2022-08-16

**Authors:** Cailong Deng, Shiyu Chen, Yong Zhang, Qixin Zhang, Feiyan Chen

**Affiliations:** 1 School of Remote Sensing and Information Engineering, Wuhan University, Wuhan 430079, China; 2 School of Geographic Sciences, Xinyang Normal University, Xinyang 464000, China; 3 Henan Engineering Research Center for Big Data of Remote Sensing and Intelligent Analysis in Huaihe River Basin, Xinyang Normal University, Xinyang 464000, China; 4 Key Laboratory for National Geographic Census and Monitoring, National Administration of Surveying, Mapping and Geoinformation, Wuhan University, Wuhan 430079, China; 5 Visiontek Research, 6 Phoenix Avenue, Wuhan 430205, China; 6 School of Electronics and Information Engineering, Wuzhou University, Wuzhou 543003, China

**Keywords:** unsupervised learning, mismatch removal, reinforcement learning, policy gradient, expected reward

## Abstract

Due to radiometric and geometric distortions between images, mismatches are inevitable. Thus, a mismatch removal process is required for improving matching accuracy. Although deep learning methods have been proved to outperform handcraft methods in specific scenarios, including image identification and point cloud classification, most learning methods are supervised and are susceptible to incorrect labeling, and labeling data is a time-consuming task. This paper takes advantage of deep reinforcement leaning (DRL) and proposes a framework named unsupervised learning for mismatch removal (ULMR). Resorting to DRL, ULMR firstly scores each state–action pair guided by the output of classification network; then, it calculates the policy gradient of the expected reward; finally, through maximizing the expected reward of state–action pairings, the optimal network can be obtained. Compared to supervised learning methods (e.g., NM-Net and LFGC), unsupervised learning methods (e.g., ULCM), and handcraft methods (e.g., RANSAC, GMS), ULMR can obtain higher precision, more remaining correct matches, and fewer remaining false matches in testing experiments. Moreover, ULMR shows greater stability, better accuracy, and higher quality in application experiments, demonstrating reduced sampling times and higher compatibility with other classification networks in ablation experiments, indicating its great potential for further use.

## 1. Introduction

Obtaining reliable matching points between image pairs is one of the core tasks in the field of computer vision and photogrammetry [1]. However, inevitable geometric and radiometric heterogenization between images results in considerable mismatches [2], which reduce the reliability of matching results and eventually lower the accuracy of vision tasks, including image fusion, change detection, 3D reconstruction, and aerial triangulation [3]. Thus, a preprocessing procedure for mismatch removal should be applied to the initial matching results for improving reliability and accuracy. Based on the approach to obtaining the optimal transformation model for removing mismatches, existing research can be generally divided into two classes: handcrafted methods and deep-learning-based methods.

Handcrafted methods iteratively acquire transformation models with global or local invariance between matched points [4]. Random sample consensus (RANSAC) [5] is one of the most representative handcrafted methods for mismatch removal. It calculates an optimal global model to constrain matching points by iteratively samplings, and has three main steps: (1) randomly sampling a minimal point pair set from the initial point pair set and computing the global geometric model (e.g., a fundamental matrix) based on a geometric solver (e.g., eight-point algorithm [6,7]); (2) estimating the point pairs in accordance with the computed model to construct a consensus set; (3) iteratively sampling a minimal set and constructing a consensus set in step (1) and (2) until a predefined threshold of sampling times is reached, and the correct match set is the maximal consensus, i.e., the subset that contains the most point pairs. The subsequent RANSAC-based methods, such as maximum likelihood estimation sample consensus (MLESAC) [8], progressive sample consensus (PROSAC) [9], differentiable RANSAC (DSAC) [10], marginalizing sample consensus (MAGSAC++) [11], and graph-cut RANSAC (GC-RANSAC) [12], aim to give a precise estimation of the probability distribution of matching inliers (i.e., correct matches) and reduce sampling times. Nevertheless, the number of samplings increases with the matching outlier rate, and RANSAC-based methods cannot find an outlier-free consensus set within a certain sampling number when the outlier rate is beyond a critical value [9,13].

Apart from RANSAC-based methods, other handcrafted methods are usually based on the assumption that local structures between images do not vary freely, due to physical constraints [14,15,16,17,18]. Thus, the local transformation model between neighbor matches can be approximated by a set of functions, and matching outliers are the point pairs that do not obey the local model [18,19,20]. Locality preserving matching (LPM) [16], grid-based motion statistics (GMS) [17], and neighborhood manifold representation consensus (NMRC) [18] are three typical locality-based methods. Specifically, LPM relaxes the geometric model by the regularized Euclidean distance; GMS gives a more simplified approximation than LPM, utilizing the number of neighbors to separate matching inliers from outliers; NMRC preserves the local neighborhood structures of matching inliers by low-dimension manifold, and uses iterative filtering based on neighborhood similarity to filter out outliers. However, these locality-based methods cannot obtain reliable matches, or even any matches, when the number of matching points is small or matching outliers are not randomly distributed, since locality-based methods are essentially statistics-based methods.

In recent years, deep-learning-based methods have paved a new way to solve the mismatch removal problem, and commonly outperform handcrafted methods [4,21,22]. Essentially, the mismatch removal problem is a binary classification problem. Deep-learning-based methods mine implicitly global or local information between matched points and classify the matching points as correct matches or mismatches based on deep neural networks (DNNs) [23,24]. If a pair of matching points is considered as a four-dimensional point, the initial matches form a set of four-dimensional point clouds, and networks with permutation invariance (such as PointNet [25] and PointNet++ [26]) can be used to separate correct matches from mismatches. To improve the stability and generalization, techniques for regularization and normalization [27,28,29,30,31] can be used in DNNs to better mine global or local information to classify the point clouds. For example, learning to find good correspondences (LFGC) [21] obtains global information by processing each point independently within a strategy of context normalization, and simultaneously minimizes the classification loss and fundamental matrix regression loss to optimize the classification network. The neighbor mining network (NM-Net) [22] mines *k* nearest compatible neighbors through a grouping module, and applies a ResNet block containing instance normalization [27] and batch normalization [28] to generate global and local features for classification and regression. The coordinate embedding network (CE-Net) [23] applies an attention mechanism [32] to aggregate global and local geometric information from matching inliers while ignoring matching outliers. Attentive context networks (ACNe) [33] combine local and global attention mechanisms and use attentive context normalization to learn the fundamental matrix for removing mismatches. There are sometimes numerous network parameters in these supervised methods, and cconsiderable labeled training data is required to train the networks to avoid overfitting [34]. Labeling data is a time-consuming task, and label errors are usually present. Erroneous labels lower performance, and in turn, futher labeled training data is required to improve the performance [35,36].

The labeling problem can be tackled by training a DNN in an unsupervised manner. Specifically, a classification network can first be used to output the matching probabilities of point pairs without labels, with the summation of these probabilities indicating the number of correct matches; then, the optimal network can be obtained by maximizing the summation, and can be used to separate matching inliers from outliers. Unsupervised learning of consensus maximization (ULCM) [37] trains PointNet by learning the maximization of the above summation with a regularization term. Due to the unsmooth loss function, ULCM can only be trained in matching sets with a constant outlier rate. Neural-guided RANSAC (NGRANSAC) [13] maximizes the expectation number of correct matches and can be trained in matching sets with arbitrary outlier rates; the expectation is differentiable if the probability distribution function (PDF) of matching inliers is continuous [38,39]. However, NGRANSAC still needs a supervised pre-training process to guarantee and speed up convergence, which leaves NGRANSAC not completely immune to erroneous labels.

The above problems of deep-learning-based methods can be handled by developing an unsupervised learning mode to train a network to separate matching outliers from inliers. Solving the mismatch removal problem can be viewed as playing a video game, where deep reinforcement learning (DRL) can be applied and surpass human performance [40,41]. Unsupervised learning can be easily implemented within the framework of DRL [42,43]. From the perspective of DRL, the classification network outputs a “policy”, and the best “policy” can guide the “player” to take actions to obtain the largest number of correct matches from the initial matching point set. Nevertheless, several issues should be addressed before DRL can be applied to train a classification network in an unsupervised manner: firstly, the video game screens evolve with the game state and player actions, whereas the initial matching sets remain constant; secondly, the game system automatically generates the reward for a player action in a particular game state (named a state–action pair), whereas there is no such reliable scoring system for the state–action pair guided by the classification network in the mismatch removal problem.

To achieve the unsupervised classification of matching outliers and inliers, we propose a framework named unsupervised learning for mismatch removal (ULMR), from the perspective of DRL. The proposed method has four main merits: (1) ULMR is unsupervised and outperforms handcrafted methods (such as RANSAC and GMS), supervised learning methods (such as LFGC and NM-Net), and the unsupervised method (ULCM) (Section 3.3 and Section 3.4); (2) ULMR can fill the learning gap to play games and solve the mismatch removal problem via DRL, i.e., it maximizes the expected reward of a state–action pair rather than that of an episode (Section 2.1, Section 2.2 and Section 2.3); (3) ULMR presents a reliable way to score every state–action pair guided by the classification network, the scoring process is unsupervised and does not require labels of training data (Section 2.4); (4) ULMR is a universal unsupervised learning framework for mismatch removal, having high compatibility with different classification networks including PointNet, NM-Net, and LFGC-Net (Section 3.5). Overall, within the framework of DRL, ULMR can train and optimize different classification networks in an unsupervised manner, directly based on the initial matching point set without labels and with different outlier rates; ULMR can extract more high quality correct matches from initial matches via fewer samplings.

## 2. Methodology

Within the framework of DRL, a DNN can be trained in an unsupervised manner to maximize the accumulated reward of state–action pairs. Inspired by the reward mechanism of DRL, we aimed to develop an unsupervised learning framework for solving the mismatch removal problem. In this section, the basic framework of DRL is firstly presented through learning to play games (Section 2.1); then, the principle of learning to remove mismatches via DRL is illustrated and analyzed (Section 2.2); finally, within the framework of DRL, we give a detailed description of sampling subsets by the Monte Carlo method (Section 2.3) and scoring of the sampled subsets (Section 2.4) for unsupervised learning of mismatch removal.

### 2.1. Basic Framework of DRL

**Figure 1:**
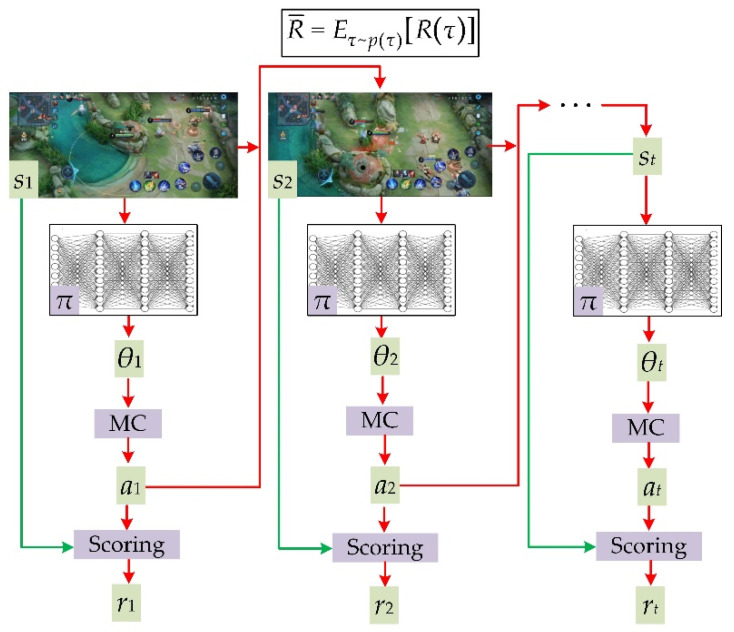
Schematic plot of applying DRL to play a video game. MC is short for Monte Carlo sampling, *r_t_* is the reward of the state–action pair (*a_t_*|*s_t_*), the box at the top shows the objective function (Equation (3)).

Unsupervised learning for playing games is a classic application of DRL [40,41]. DRL generally consists of four basic ingredients in the process of training a deep network to play games (as shown in Figure 1): the game state *s* generated by the game system, the game policy *θ* generated by a DNN *π*, the action *a* is guided by the game policy *θ* and the reward *r* of the state–action pair (*a*|*s*).

In the general case of learning to play games via DRL, training data are the game states. The current game state *s_t_* will be input into the game policy DNN *π*, and the network *π* will output a game policy *θ_t_*, namely *θ_t_* = *π*(*s_t_*;*ω*), where *ω* are parameters of network *π*. Then the “player” (sampling methods sometimes act as the “player”) takes action *a_t_* with the given state *s_t_* under the guidance of game policy *θ_t_*. Subsequently, the game system gives a reward *r_t_* for the state–action pair (*a_t_*|*s_t_*), and then the game enters a new state *s_t_*_+1_. As the process evolves, an episode *τ* = {(*a*_1_|*s*_1_),(*a*_2_|*s*_2_),…,(*a_t_*|*s_t_*)} and the accumulated reward of *τ* can be obtained:

(1) $$R(\tau )=\sum_{t=1}^{T}r(a_{t}|s_{t})$$

where *R*(*τ*) is the accumulated reward, *T* is the total number of state–action pairs in an episode *τ*, *r*(*a_t_*|*s_t_*) is the reward of the state–action pair (*a_t_*|*s_t_*).

Intuitively, the optimal game policy network parameters *ω* can be obtained by maximizing *R*(*τ*). While the episode *τ* involves a sampling process such as Monte Carlo (MC) sampling [44], the gradient of *R*(*τ*) with respect to the game policy *θ_t_* is invalid. Therefore, the gradient-based methods cannot be used directly to maximize *R*(*τ*). Alternatively, we can treat the episode *τ* as a randomly generated sequence and maximize the expected reward of *τ*, since the gradient of the expected reward is valid while the PDF of *τ* is a continuous function of *θ_t_* [38]. Next, we will show that *p*(*τ*) is essentially a continuous function of *θ_t_*.

It can be assumed that the current game state *s_t_*_+1_ only depends on *s_t_* and is independent of *s_t_*_−1._ the PDF of episode *τ* can be described by a Markov decision process [45]:

(2) $$p(\tau )=p(s_{1})\prod_{t=1}^{T}p(a_{t}|s_{t};\theta_{t})p(s_{t+1}|s_{t},a_{t})$$

where *p*(*s*_1_) is the probability of state *s*_1_ when taking no actions, *p*(*a_t_*|*s_t_*;*θ_t_*) is the probability of the state–action pair (*a_t_*|*s_t_*) under the guidance of game policy *θ_t_*, and *p*(*s_t_*_+1_|*s_t_*,*a_t_*) is the state transition probability from *s_t_* to *s_t_*_+1_ under the condition of action *a_t_*. Moreover, the game policy *θ_t_* is the parameter of probability distribution *p*(*a_t_*|*s_t_*;*θ_t_*), thus *p*(*τ*) is a function of *θ_t_*. Note that *p*(*s_t_*_+1_|*s_t_*,*a_t_*) and *p*(*s*_1_) are determined by the game system and independent of *θ_t_*, and *θ_t_* is a continuous output of DNN *π*. Thus, *p*(*τ*) is a continuous function of *θ_t_*, and the gradient of the expected reward is valid. Given Equation (2), the expected reward of an episode *τ* can be expressed as:

(3) $$\bar{R}=E_{\tau \sim p(\tau )}[R(\tau )]=\sum_{t=1}^{T}E_{\left(a_{t}|s_{t})\sim p(a_{t}|s_{t};\theta_{t}\right)}(r(a_{t}|s_{t}))$$

where $\bar{R}$ is the expected reward of an episode; *τ*~*p*(*τ*) means sampling an episode from *p*(*τ*); and (*a_t_*|*s_t_*)~*p*(*a_t_*|*s_t_*;*θ_t_*) means sampling a state–action pair (*a_t_*|*s_t_*) from *p*(*a_t_*|*s_t_*;*θ_t_*). To maximize the objective function Equation (3), we should calculate the gradient of the expected reward $\bar{R}$ with respect to *θ_t_* (named as policy gradient) [46]:

(4) $$\frac{\partial \bar{R}}{\partial \theta_{t}}=\sum_{t=1}^{T}E_{a_{t}\sim p_{\theta_{t}}}(r_{t}\frac{\partial \log p_{\theta_{t}}}{\partial \theta_{t}})$$

where *a_t_*, $p_{\theta_{t}}$, and *r_t_* are short for (*a_t_*|*s_t_*), *p*(*a_t_*|*s_t_*;*θ_t_*), and *r*(*a_t_*|*s_t_*), respectively. 

Based on the basic framework of DRL (Figure 1), we aimed to train a DNN for mismatch removal in an unsupervised way. The analogies and differences between playing games and removing mismatches are specified in the next section.

### 2.2. Learning to Remove Mismatches

**Figure 2:**
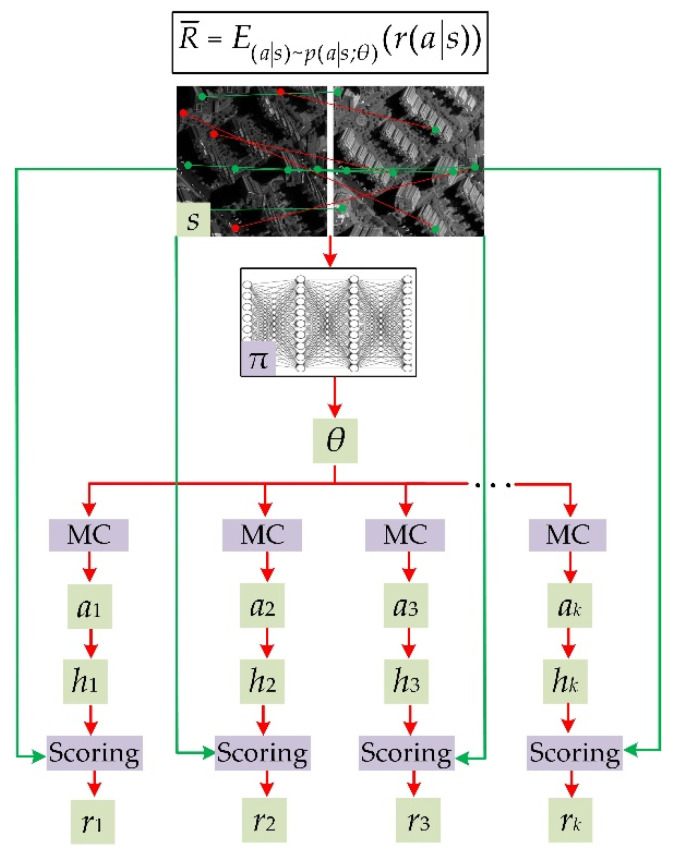
Schematic plot of learning to remove mismatches via DRL. The objective function (Equation (5)) is shown in the box at the top.

DRL can be applied to solve the mismatch removal problem. As illustrated in Figure 2, the state *s* can be viewed as the initial matched point pair set between stereo images; the policy DNN *π* is a classification network which outputs a relative matching probability (RMP) for every matched point pair (RMP is analogous to the game policy *θ*, here we continue to use *θ* to denote RMP); action *a_k_* means sampling a subset from the matched point pair set *s*, which is used to generate a hypothesis *h_k_*; reward *r_k_* is the score of the state–action pair (*a_k_*|*s*) based on the hypothesis *h_k_*. The hypothesis is that matching inliers conform to the transformation model, which was calculated via the sampled subset and a geometric model solver (e.g., the fundamental matrix computed by the eight-point algorithm), while matching outliers were not in accordance with the model.

Although the above-mentioned analogies are intuitive, there still exist some differences between learning to remove mismatches and learning to play a video game. As shown in Figure 1, the current state *s_t_*_+1_ is conditioned by the last state–action pair (*a_t_*|*s_t_*), thus the game states evolved in the training. As shown in Figure 2, in the process of learning to remove mismatches, the state *s* was the initial matching point set which remained unchanged regardless of every action. Thus, the number of state–action pairs in an episode *τ* is 1, i.e., *T* = 1 in Equations (1) and (2); when applying DRL to remove mismatches, it worked to maximize the expected reward of a state–action pair rather than that of an episode. The objective function in Equation (3) and the policy gradient in Equation (4) turn into:

(5) $$\bar{R}=E_{\left(a|s)\sim p(a|s;\theta \right)}(r(a|s))$$

(6) $$\frac{\partial \bar{R}}{\partial \theta }=E_{a\sim p_{\theta }}(r\frac{\partial \log p_{\theta }}{\partial \theta })$$

where *a*, *p_θ_*, *r* in Equation (6) is short for the state–action pair (*a*|*s*), the probability distribution function (PDF) *p*(*a*|*s*;*θ*), and reward *r*(*a*|*s*), respectively; *a*~*p_θ_* means that action *a* is taken from the distribution *p*(*a*|*s*;*θ*).

The policy gradient $\frac{\partial \bar{R}}{\partial \theta }$ in Equation (6) is the key for learning to remove mismatches. Next will be presented the detailed processes of calculating the policy gradient, i.e., (1) computing probability distribution *p_θ_* and sampling subsets from *p_θ_*, and (2) calculating the reward *r* of the sampled subset and approximating the expectation by the mean.

### 2.3. Sampling Subsets by Monte Carlo

As mentioned in the above analogies, state–action pair (*a*|*s*) is the action *a* of sampling a subset from matched point pair set *s*, with elements of the subset drawn from the distribution *p*(*a*|*s*;*θ*). The problem of sampling a subset from a given distribution can be addressed by MC sampling. As shown in Figure 2, the classification network *π* outputs RMPs (namely a vector *θ* = (*θ*^1^, *θ*^2^, …, *θ^n^*)) for the matching point pairs in the set *s*, and a subset is drawn from the categorical distribution parametrized by *θ* [47]:

(7) $$Cat(y_{i}|\theta )=\theta^{i}$$

where *y_i_* is the *i*’th matching point pair in set *s*; *θ^i^* is the *i*’th element in *θ* = (*θ*^1^, *θ*^2^, …, *θ^n^*), meaning the probability of sampling point pair *y_i_* from set *s*. With an independently identical distributed assumption, the sampled subset Ω can be obtained from the PDF *p_θ_*:

(8) $$p_{\theta }=\overset{m}{\underset{j=1}{\Pi }}Cat(y_{j}|\theta )=\overset{m}{\underset{j=1}{\Pi }}\theta^{I(y_{j})}$$

where *m* is the number of elements in the sampled subset Ω; *y_j_* is a matched point pair in the subset Ω; I(*y_j_*) is the operation of obtaining the index of *y_j_* in the initial matching point set *s*.

When PDF *p_θ_* has been determined, MC can be applied to sample a subset Ω. The sampling algorithm is illustrated in Algorithm 1. At the beginning of the sampling, there was no prior information for the matching point pairs, and the classification network output an approximately equal RMP for every matched point pair. Therefore, each matching pair had an equal chance of being chosen (in this case, the sampling process is similar to RANSAC); as the training progresses, the classification network outputs higher RMPs for matching inliers than mismatches, thus, matching inliers become prone to being sampled; ultimately, Algorithm 1 can sample a subset containing *m* correct matches. Thus, the proposed sampling algorithm can speed up training of the classification network.
**Algorithm 1:** Sampling a minimal subset Ω containing *m* pairs of matched points**Input:** A point pair set *s* containing *n* pairs of points, and the RMPs *θ* (Equation (7)) 
**Output:** A sampled subset with the PDF *p_θ_* (Equation (8)) 
1 Initialize Ω as an empty list
**for** *j* = 1 to *m*

 2 Draw independent random variables *u*_1_,…,*u_n_* from uniform distribution *U*(0,1) 
 3 Select one point pair *y_j_* with index *i* in set *s*, 
  where $i=\underset{1\leq i\leq n}{\mathrm{argmax}}[\log(u_{i}/\left(1-u_{i}\right))-\mathrm{loglog}(1/u_{i})]$

 4 If *y_j_* is already in Ω, repeat step 2 and 3 until a new point pair is selected 
 5 Append *y_j_* to Ω
**End**6 Return Ω

### 2.4. Scoring a Sampled Subset

As shown in Figure 1, the game system automatically generates scores for state–action pairs, whereas there is no such a reliable scoring system for state–action pairs in the mismatch removal problem. Once the sampled subset Ω has been obtained, the reward *r* of a state–action pair (*a*|*s*) based on the corresponding hypothesis can be given as follows. Firstly, a transformation model (e.g., fundamental matrix, essential matrix, or homography matrix) is calculated based on the sampled subset Ω. Then the corresponding consensus set *C* can be obtained by collecting the matching point pairs within a predefined back-projective error threshold. Finally, the element number of consensus set *C* is the reward of the state–action pair (*a*|*s*):

(9) r=|C|

where |⋅| is an element counter of a set. As the scoring process does not require labels of training data, the process is unsupervised.

In general, the expectation shown in Equations (5) and (6) can be approximated by the mean if there are plenty of samples. Algorithm 1 can be repeated *N* times to generate *N* subsets, and the mean of the *N* sampled subsets can be utilized to approximate the expectation. Therefore, Equation (6) can be computed as:

(10) $$\frac{\partial \bar{R}}{\partial \theta }=\frac{1}{N}\sum_{k=1}^{N}r_{k}\frac{\partial (\sum_{j=1}^{m}\log\theta^{I(y_{j}^{\left(k\right)})})}{\partial \theta }=\frac{1}{N}\sum_{k=1}^{N}(r_{k}\times \sum_{j=1}^{m}\frac{1}{\theta^{I(y_{j}^{\left(k\right)})}}\mathrm{one}\_\mathrm{hot}(I(y_{j}^{\left(k\right)}))$$

where *r_k_* is the reward of the *k*’th sampled subset Ω*_k_*; $y_{j}^{\left(k\right)}$ is the *j*’th matching point pair in the subset Ω*_k_*; $I(y_{j}^{\left(k\right)})$ is the operation of obtaining the index of $y_{j}^{\left(k\right)}$ in matching point set *s*, and $\theta^{I(y_{j}^{\left(k\right)})}$ is the RMP of $y_{j}^{\left(k\right)}$; one_hot(⋅) is a function that outputs a *n* dimensional vector where an element is 1 in the corresponding dimension and the rest are 0 s. Thus, the policy gradient $\frac{\partial \bar{R}}{\partial \theta }$ can be explicitly calculated by Equation (10) based on the scoring system and the PDF *p_θ_*, making the classification network easier to tune.

Once the policy gradient has been computed (i.e., Equation (10)), the gradients of $\bar{R}$ with respect to the network parameters *ω* can be computed by the chain rule:

(11) $$\frac{\partial \bar{R}}{\partial \omega }=\frac{\partial \bar{R}}{\partial \theta }\cdot \frac{\partial \theta }{\partial \omega }$$

where $\frac{\partial \theta }{\partial \omega }$ is the gradient of RMPs with respect to *ω*, and can be obtained by a deep learning framework such as Pytorch [48] or Tensorflow [49]. Finally, the network parameters can be optimized in the training iterations:

(12) $$\omega^{\left(i+1\right)}=\omega^{\left(i\right)}+\eta \times \frac{\partial \bar{R}}{\partial \omega^{\left(i\right)}}$$

where *i* is the *i*’th iteration; η is the learning rate; $\frac{\partial \bar{R}}{\partial \omega^{\left(i\right)}}$ is the gradient of $\bar{R}$ w.r.t. $\omega^{\left(i\right)}$. Equation (12) can be calculated by built-in framework methods such as stochastic gradient descent (SGD) [50] and Adam [51]. Note that SGD and Adam achieve minimization, and we should apply an opposite number for the reward *r*(*a*|*s*) when using the two methods to maximize Equation (5).

## 3. Experiments

To evaluate the proposed ULMR, its implementation details are given first (Section 3.1), followed by the compared benchmark algorithms and the training data (Section 3.2). Then, these algorithms are rated in test experiments (Section 3.3) and application experiments (Section 3.4). Finally, an ablation experiment was conducted for further testing of ULMR (Section 3.5). 

### 3.1. Implementation Details

#### 3.1.1. Network Architecture

Theoretically, the proposed ULMR can be integrated with different classification networks that have the merit of permutation invariance. For widely-used classification networks such as PointNet, NM-Net, and LFGC-Net, source codes are available. We found in our experiments that NM-Net performed slightly better in recall and precision (as shown in Section 3.5) than the other two networks. Since recall and precision are the two key parameters for the evaluation of mismatch removal, we choose NM-Net as the classification network in ULMR.

**Figure 3:**
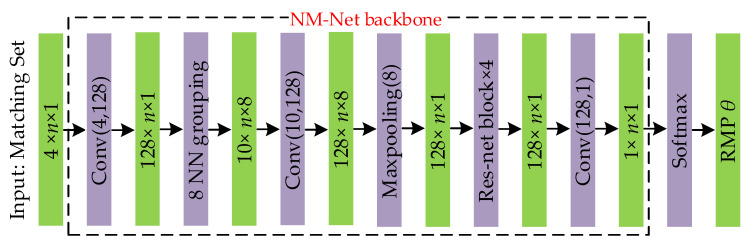
Network architecture used in ULMR. Conv(*i*, *o*) represents a convolutional operation with additional operations such as batch normalization and activation, the input data has *i* channels, and the output data has *o* channels; Res-Net block × 4 represents four residual connected convolutional networks; Maxpooling(*i*) represents a max pooling operation with inputted data that has *i* channels; and 8NN grouping mines eight nearest neighbors’ information in a hierarchical structure.

We added a softmax operation after NM-Net, as the network *π* is expected to output RMP for each matched point pair; the detailed network architecture used in ULMR is shown in Figure 3. For a matched point pair set that consists of *n* matched points, the network output an *n*-dimensional vector *θ* = (*θ*^1^, *θ*^2^, …, *θ^n^*) indicating the matching reliability. Note that ULMR is a universal framework for unsupervised learning of mismatch removal, and it can be integrated with other classification networks such as PointNet or LFGC, which can be seen in the ablation experiments.

#### 3.1.2. Training and Predicting Pipelines

**Figure 4:**
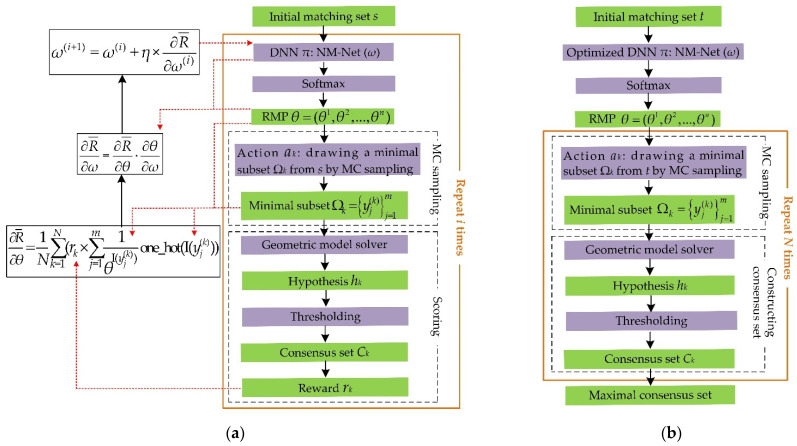
Training and predicting pipelines of ULMR. (**a**) Training pipeline, (**b**) Predicting pipeline.

The training pipeline of ULMR is shown in Figure 4a: the input was the initial matching set without labels, and NM-Net outputs RMP for every matching pair; then, depending on the RMPs, the MC algorithm (Algorithm 1) was applied to sample a subset consisting of *m* point pairs from a categorical distribution parameterized by RMPs (Equations (7) and (8)); next, the sampled subset was fed to a geometric model solver to generate a hypothesis; finally, by thresholding the back-projective error (e.g., symmetric epipolar error), we obtained a consensus set, with the element number of the consensus set as the reward (Equation (9)). In the above process, Equations (10)–(12) can be computed, and the optimal parameters *ω* of NM-Net can be obtained by the training iterations. Note that the optimal NM-Net was not used directly to distinguish outliers, since there is no reliable threshold of RMP to separate inliers from outliers. As depicted in Figure 4b, based on the optimized NM-Net, Algorithm 1 was repeated *N* times to generate *N* subsets and estimate their corresponding hypotheses; then, *N* consensus sets were obtained based on the thresholding; finally, the maximal consensus set with the most elements was considered as the outlier free matching set.

#### 3.1.3. Training Settings

We applied the epipolar constraint [52] to separate matching inliers from outliers, and used the eight-point algorithm [6,7] as the geometric model solver to estimate hypotheses of the fundamental matrices. It was necessary to sample a minimal subset consisting of eight matched point pairs each time (namely *m* = 8). The epipolar error threshold [39] for estimating consensus sets was 3.0 pixels. The sampling number *N* in Equation (10) was 100. The training batch size was 32, the training number of epochs was 60, the optimizer was SGD [50], and the initial learning rate was 0.01 with a cosine annealing learning rate decay, in which the period was 30 and the minimum learning rate was 0.

### 3.2. Benchmark Algorithms and Training Data

#### 3.2.1. Benchmark Algorithms

We compared ULMR to handcrafted methods (RANSAC and GMS), supervised methods (LFGC and NM-Net), and an unsupervised method (ULCM). All the deep learning methods were trained with the same dataset as ULMR. The key parameter settings of the compared methods are listed in Table 1.

#### 3.2.2. Training Data

The training data were brown_bm_1 in the SUN3D database [53] and st_peters_square in the Yahoo YFCC100M database [54]. For supervised methods, we firstly extracted and matched 2000 image keypoints for every image pair, using scale-invariant feature transform (SIFT) [55]. Then, the structure from motion method (implemented in COLMAP [56]) was applied to obtain the pose for each image, and the accurate fundamental matrices were estimated with the camera matrices. Finally, we labeled the matches with symmetric epipolar errors smaller than 3.0 pixels as inliers, and the rest of the matches as outliers [21,22]. For ULCM, we calculated the inlier rate for the batched training point pairs, and for our proposed URML, we labeled nothing and merely used the matched SIFT keypoints for training.

### 3.3. Test Experiments of Real Scenario Images

**Figure 5:**
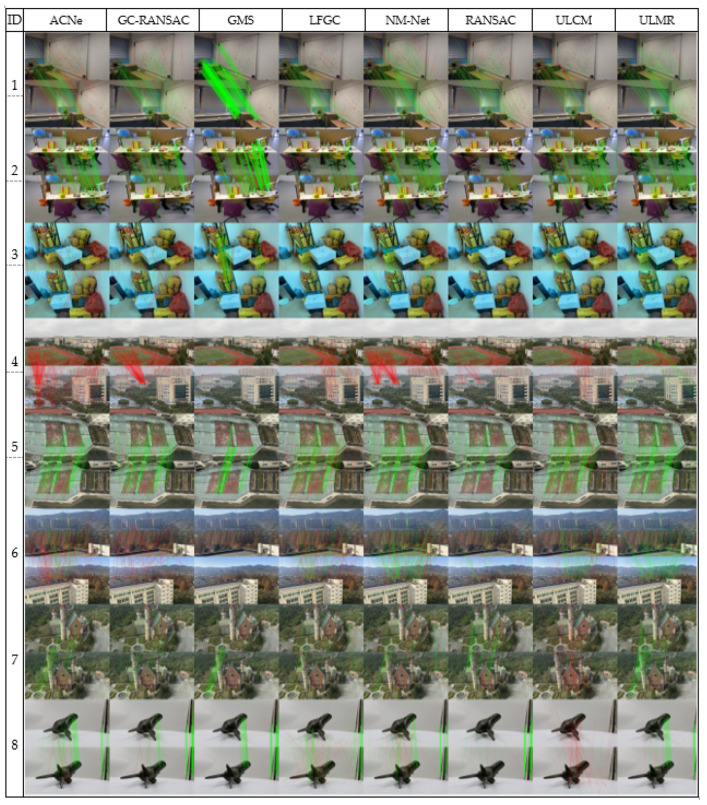
The mismatch removal results of real scenario images. By manually checking, RCM and RFM are connected by green and red lines, respectively.

Eight pairs of real scenario images were collected as the test dataset. As shown in Figure 5, pairs one–three depicted indoor scenes with significant viewpoint changes, and pairs four–seven were outdoor images with simultaneous viewpoint and scale changes; furthermore, image pair seven was from an unmanned aerial vehicle (UAV), and pair eight was sampled from macrophotography. Except for image pairs one and two from Brachmann and Rother [13], and pairs seven and eight from the GL3d dataset [57], the other image pairs were captured by mobile phone cameras.

**Figure 6:**
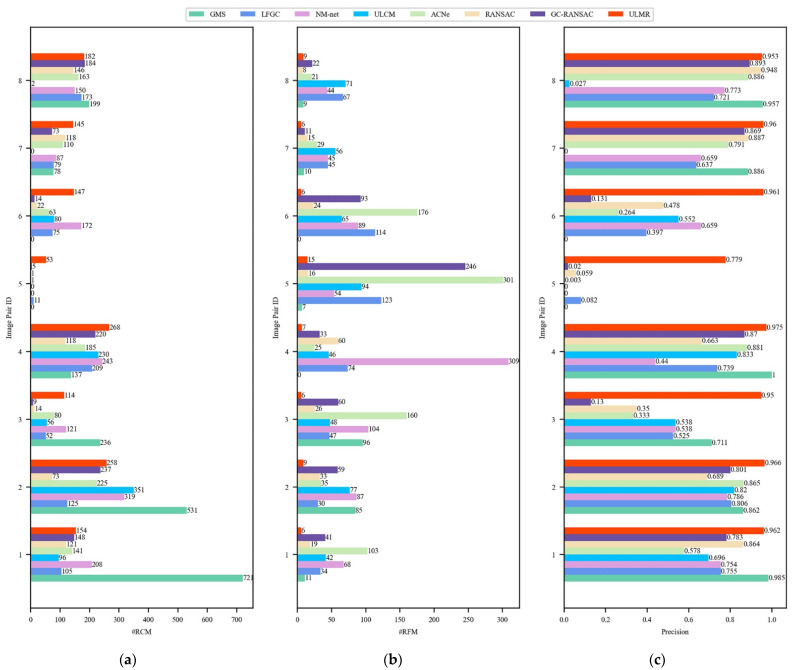
Quantitative mismatch removal results of real scenario images. (**a**) #Precision; (**b**) #RCM; (**c**) #RFM.

The initial matches were obtained by SIFT (ratio testing [55] was not applied), the number of initial matches in every image pair was 2000, the mean outlier rate of initial matches was higher than 80%. The mismatch removal results for these methods are presented visually in Figure 5, and the number of remaining correct matches (#RCM), number of remaining false matches (#RFM), and precision (#RCM divided by the number of remaining matches) are estimated in Figure 6.

Figure 5 and Figure 6 visually demonstrate that the proposed ULMR obtained better precision and smaller #RFM than all the compared methods, and the RCMs were sufficient and evenly distributed. First, ULMR achieved the best precision among the above methods; as shown in Figure 6a, the mean precision of ULMR was close to 93.8% and the lowest precision was still higher than 77.9%. Second, ULMR obtained the smallest #RFM. The mean #RFM of ULMR was 8.0 and the largest #RFM was less than 15 (shown Figure 6c). Third, though ULMR did not obtain the most #RCM in all experimental image pairs, the #RCM of ULMR were all beyond 50 (shown Figure 6b), sufficient for particular applications such as self-localization and aerotriangulation in photogrammetry. Furthermore, the even distribution of ULMR’s RCM (shown in Figure 5) can make a contribution to improving positional accuracy [3].

The better performance of ULMR can be mainly attributed to the merits derived from RANSAC, i.e., finding the maximal consensus set by sampling. Meanwhile, ULMR amends RANSAC’s uniform sampling to policy-guided sampling. The classification network used in ULMR outputs higher RPMs for the matching inliers, which guarantees ULMR’s production of good subsets for estimating precise fundamental matrices. The precise fundamental matrices can promote the aggregation of maximum consensus sets. Consequently, ULMR can obtain better experimental results when the mean outlier rate is higher than 80%. RANSAC samples each matching inlier and outlier with equal probability, and it cannot process the initial matches with a high outlier rate (e.g., higher than 80%). Although GC-RANSAC considers the spatial coherent structures of the matched points, its improvements are limited compared to RANSAC.

GMS obtained the largest #RCM and the most desirable precision only in image pair one with structured textures. Structured textures contain aggregated matching point pairs which facilitate the precise estimation of motion; thus, GMS can separate outliers from inliers. When there are insufficient structured textures, the performance of GMS is degradative. For example, in image pairs five and six, all the matches have been filtered out and no inlier is left.

The supervised methods, namely LFGC, NM-Net, and ACNe, also performed worse than the proposed ULMR. Though the labeling method presented above is relatively accurate and widely-used [21,22], labeling errors are inevitable as the epipolar constraint is necessary but insufficient to separate all inliers from outliers. Label errors degrade stability and result in poor performance of supervised methods. For the unsupervised method used in ULCM, the inlier rate of batched training data is still required in advance. Essentially, inlier rate labeling for training data also needs to classify matching inliers and outliers, thus ULCM encounters the same issue as supervised methods.

### 3.4. Application Experiments of Real Tasks

The real task data was the Reichstag dataset containing 1174 image pairs in the Yahoo YFCC100M dataset [54]; these images contain considerable changes of viewpoints, illuminations, and scales. We applied the proposed ULMR to estimate the fundamental matrix and compared the results with GMS, RANSAC, GC-RANSAC, NM-Net, LFGC, ACNe, and ULCM. The workflow of the application experiments was as follows: firstly, SIFT was adopted to extract 2000 initial matching points between each image pair without ratio test [55], and the average outlier rate of initial matches was higher than 80%; then, the above mismatch removal methods were applied to purify the initial matches; next, the eight-point algorithm was applied to estimate fundamental matrices between the image pairs; finally, we used positional accuracy to evaluate the performances of the methods.

For GMS, ULCM, NM-Net, LFGC, and ACNe, their purified results had lower precision and numerous matching outliers (as shown in Section 3.3), causing degenerated configuration when using eight-point algorithm to estimate fundamental matrices. Therefore, a RANSAC-embedded eight-point algorithm was applied instead. Nevertheless, we used pure ULMR for the application.

**Figure 7:**
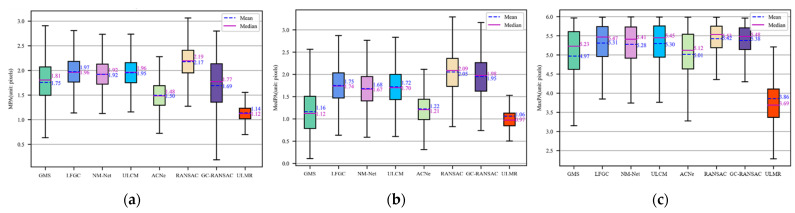
Box plots of positional accuracy for the remaining matches. (**a**) MPA; (**b**) MedMPA; (**c**) MaxPA.

For each image pair with sufficient and valid matches after mismatch removal, we estimated their MPA (mean positional accuracy), MaxPA (max positional accuracy), and MedPA (median positional accuracy):

(13) $$\begin{array}{l} \mathrm{MPA}=\sqrt{(\sum_{i=1}^{nA}d(y_{i},h))/nA}\\ \mathrm{MaxPA}=\max(\{d(y_{i},h)|1\leq i\leq nA\})\\ \mathrm{MedPA}=\mathrm{median}(\{d(y_{i},h)|1\leq i\leq nA\}) \end{array}$$

where *d*(*y_i_*,*h*) is the symmetric epipolar error of matched point pair *y_i_* with given fundamental matrix *h*; *nA* is the number of remaining matches in an image pair; max({⋅}) and median({⋅}) are the operators of obtaining the max and median elements from a set. MPA gives the overall accuracy of the remaining matches; MaxPA shows the quality of the remaining matches, larger MaxPA means there remains at least one matching outlier with very poor positional accuracy; MedPA coupled with MPA verifies the accuracy stability of the remaining matches. The results of application experiments are shown in Figure 7.

Figure 7 shows that pure ULMR obtained the most desirable results among the above methods. First, ULMR achieved the greatest stability; as shown in Figure 7a,b, ULMR had the lowest box heights, indicating smaller fluctuations of MPA and MedPA. Second, ULMR had the best accuracy; as illustrated in Figure 7a,b, its mean MPA, median MPA, mean MedPA, and median MedPA were all the lowest. Third, the remaining matches obtained by ULMR had the highest quality; as shown in Figure 7c, the MaxPA of ULMR had the lowest upper bound (5.25 pixels), and the smallest mean MaxPA (3.69 pixels) and median MaxPA (3.86 pixels).

These RANSAC-integrated learning methods outstrip pure RANSAC; since Reichstag dataset has an outlier rate higher than 80%, it is difficult for RANSAC to process. GMS delivered higher performances than those learning-based methods apart from ULMR and ACNe, as GMS is integrated with RANSAC and Reichstag consists of images formed by building blocks with structured textures; meanwhile, ACNe performed slightly better than GMS, as ACNe can extract more geometric information for removing mismatches bases on local and global attention mechanisms.

### 3.5. Ablation Experiments

We use the same Reichstag dataset as in Section 3.4 to test the proposed ULMR in ablation experiments; and the main concerns were the effect of sampling number and the compatibility of the ULMR framework with other classification networks. We used precision, inlier recall, and outlier recall for the quantitative evaluations:

(14) $$ir=\frac{\#\mathrm{RCM}}{\#\mathrm{ICM}};\;or=1-\frac{\#\mathrm{RFM}}{\#\mathrm{IFM}}$$

where *ir* and *or* are inlier recall and outlier recall, respectively; #ICM is the number of initially correct matches; and #IFM is the number of initially false matches. Precision gives an overall evaluation of mismatch removal methods and mainly determines positional accuracy. However, precision may be biased when initial matches have a higher outlier rate, as some poor methods can consider most of matches as outliers and still achieve a high precision. Therefore, we used *ir* and *or* to compensate for the bias and give a comprehensive evaluation.

#### 3.5.1. Effect of Sampling Number

**Figure 8:**
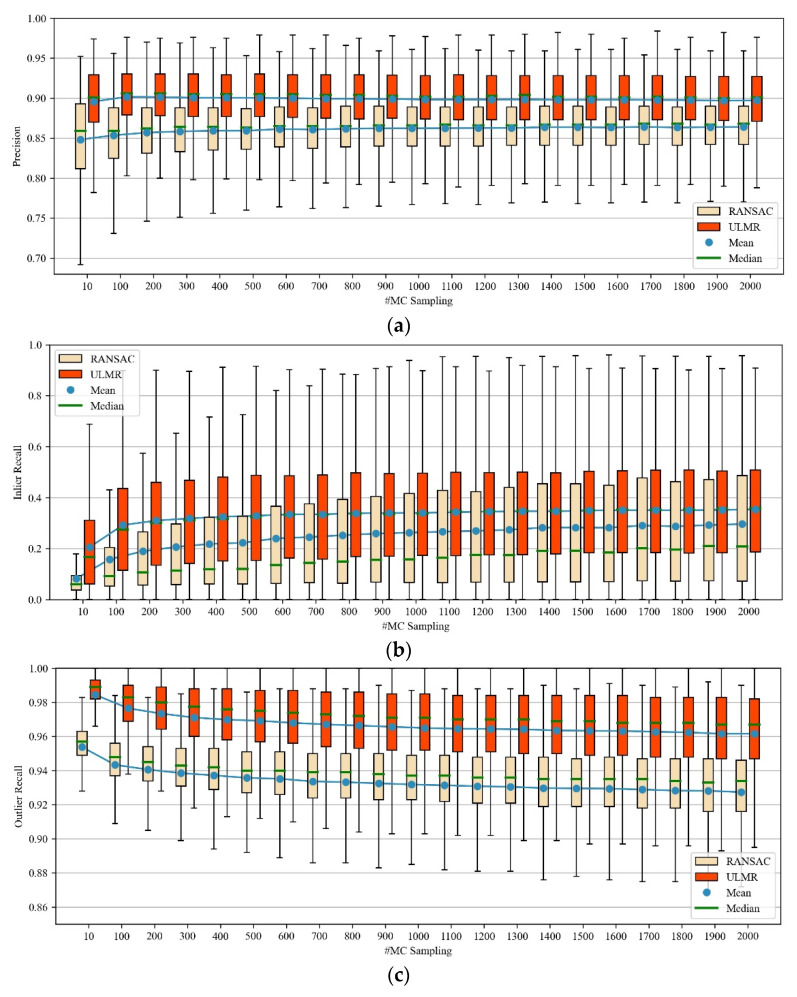
Boxplots of precision, inlier recall, and outlier recall of ULMR and RANSAC. (**a**) Precision; (**b**) inlier recall; (**c**) outlier recall.

We scrutinized how precision, inlier recall, and outlier recall varied with the sampling number; the compared results of ULMR and RANSAC are illustrated in Figure 8.

As shown in Figure 8, compared with RANSAC, ULMR drew fewer samplings while achieving better performance. When the MC sampling number was 100, ULMR achieved the highest precision (about 0.90), and the best tradeoff for outlier recall (about 0.98) and inlier recall (about 0.30). In contrast, RANSAC achieved its optimal performance with sampling number 1500, when precision, outlier recall, and inlier recall were approximately 0.87, 0.93 and 0.30, respectively. ULMR utilizes NM-Net to assign higher RMPs to correct matches, thus a good subset with all correct matches is more likely to be obtained by the sampling algorithm (i.e., Algorithm 1) from a limited number of samplings. Furthermore, these characteristics of ULMR can speed up the training of the classification network. In contrast, each matching inlier and outlier have an equivalent probability of being sampled by RANSAC, so more sampling time is required to build an outlier-free subset.

#### 3.5.2. Compatibility with Other Classification Networks

**Figure 9:**
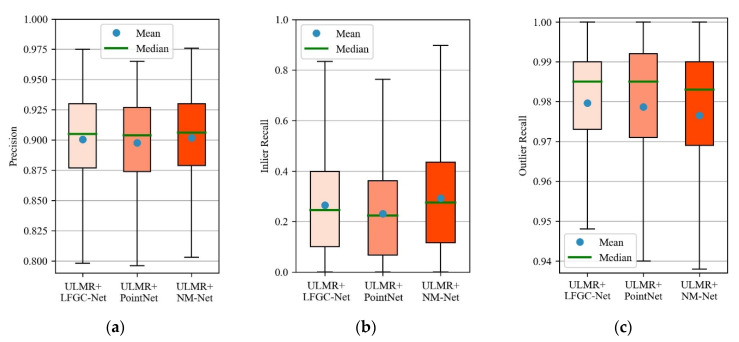
Boxplots of precision, inlier recall, and outlier recall for the three integrated methods. (**a**) Precision; (**b**) inlier recall; (**c**) outlier recall.

In Section 3.3 and Section 3.4, we combined the ULMR framework with NM-Net. Theoretically, ULMR is a universal framework that can be integrated with other classification networks, such as LFGC-Net (used in LFGC) or PointNet. We combined ULMR with NM-Net, LFGC-Net, and PointNet to obtain three integrated methods (ULMR + NM-Net, ULMR+LFGC-Net, ULMR + PointNet, respectively). Experimental results of the three methods are shown in Figure 9.

It can be seen from Figure 9 that the three integrated methods all achieved excellent precision, inlier recall, and outlier recall, which shows the high compatibility of the proposed ULMR framework with other classification networks. ULMR + NM-Net performed slightly better than ULMR + LFGC-Net and ULMR + PointNet, because NM-Net aggregates more global and local information than PointNet or LFGC-Net [22].

## 4. Conclusions

This paper proposes an unsupervised learning method for mismatch removal (named ULMR). Within the framework of DRL, ULMR was able to train networks in an unsupervised manner and successfully separate mismatches from correct matches. Test experiments of real scenario images showed that the proposed ULMR had higher precision, attaining more remaining correct matches, and fewer false matches compared with RANSAC and GMS (handcrafted methods), LFGC and NM-Net (supervised learning methods), or ULCM (unsupervised method). Moreover, in the application experiments of real tasks, ULMR obtained better positional accuracy, greater stability, and higher quality than the above compared methods. In addition, ablation experiments demonstrated that ULMR delivered more stable results with a smaller number of samplings. Meanwhile, the ULMR framework was shown to be highly compatible with widely used classification networks such as PointNet, NM-Net, and LFGC-Net. Hence, potential for practical use of our proposed ULMR can be expected. However, in order to score state–action pairs accurately and the train a classification network in an unsupervised way, the strict geometric model between image pairs must be known and determined in advance; otherwise, a reliable scoring function cannot be obtained, and expert demonstration methods [58] may be required to score state–action pairs, so ULMR becomes a supervised method.

## Figures and Tables

**Table 1:** Key parameter settings of the compared methods.

Method	Code Resource (Accessed on 1 June 2022)	Key Parameter Setting
RANSAC	https://github.com/opencv/opencv/blob/4.x/modules/calib3d/src/fundam.cpp	Epipolar error threshold: 3.0 pixels; Maximum number of iterations: 2000
GC-RANSAC	https://github.com/danini/graph-cut-ransac	As above
GMS	https://github.com/JiawangBian/GMS-Feature-Matcher	Grid size: 20 × 20; Number of neighbors: 9; with rotation: true; With scale: true
LFGC	https://github.com/vcg-uvic/learned-correspondence-release	Default as in [21]
NM-Net	https://github.com/sailor-z/NM-Net	Default as in [22]
ULCM	https://bitbucket.org/probstt/ulcm-public/src/master/	Default as in [37]
ACNe	https://github.com/vcg-uvic/acne	Inlier clustering type: combined

## Data Availability

The datasets used in this paper are public data.
